# Preparation of Nanocomposite Plasmonic Films Made from Cellulose Nanocrystals or Mesoporous Silica Decorated with Unidirectionally Aligned Gold Nanorods

**DOI:** 10.3390/ma7043021

**Published:** 2014-04-11

**Authors:** Michael G. Campbell, Qingkun Liu, Aric Sanders, Julian S. Evans, Ivan I. Smalyukh

**Affiliations:** 1 Department of Physics, University of Colorado, Boulder, CO 80309, USA; E-Mails: michael.g.campbell@colorado.edu (M.G.C.); qingkun.liu@colorado.edu (Q.L); julian.evans@colorado.edu (J.S.E.); Ivan.Smalyukh@colorado.edu (I.I.S.); 2 National Institute of Standards and Technology, Boulder, CO 80305, USA; E-Mail: aric.sanders@nist.gov; 3 Department of Electrical, Computer, and Energy Engineering, Materials Science and Engineering Program, and Liquid Crystal Materials Research Center, University of Colorado, Boulder, CO 80309, USA; 4 National Renewable Energy Laboratory, Renewable and Sustainable Energy Institute, University of Colorado, Boulder, CO 80309, USA

**Keywords:** gold nanorods, cellulose nanocrystals, liquid crystal, plasmonics, mesoporous silica

## Abstract

Using liquid crystalline self-assembly of cellulose nanocrystals, we achieve long-range alignment of anisotropic metal nanoparticles in colloidal nanocrystal dispersions that are then used to deposit thin structured films with ordering features highly dependent on the deposition method. These hybrid films are comprised of gold nanorods unidirectionally aligned in a matrix that can be made of ordered cellulose nanocrystals or silica nanostructures obtained by using cellulose-based nanostructures as a replica. The ensuing long-range alignment of gold nanorods in both cellulose-based and nanoporous silica films results in a polarization-sensitive surface plasmon resonance. The demonstrated device-scale bulk nanoparticle alignment may enable engineering of new material properties arising from combining the orientational ordering of host nanostructures and properties of the anisotropic plasmonic metal nanoparticles. Our approach may also allow for scalable fabrication of plasmonic polarizers and nanoporous silica structures with orientationally ordered anisotropic plasmonic nanoinclusions.

## 1. Introduction

Nanostructured materials are poised to revolutionize scientific instruments, technologies, and consumer devices. Liquid crystalline intermediate phases confer long-range orientational order that has been previously used to improve the properties of fibers and deposited films for biomedical [1], optical [2], and electronic [3,4] applications. Liquid crystals (LCs) can act as smart hosts that align anisotropic nanoparticle inclusions and leverage nanoscale anisotropy into device scale polarization sensitivity [5]. Self-assembly of plasmonic nanoparticles in LCs has been extensively studied recently [6–8]. Gold nanorods (GNRs) have two surface plasmon resonance (SPR) modes, the transverse mode at 525 nm and red-shifted longitudinal mode dependent on the aspect ratio, associated with short and long axes of the rod, respectively [9]. Aligning GNRs allows one to produce so-called “plasmonic polarizers”, which have been previously realized in stretched polyvinyl alcohol films [10] and magnetically tunable LC systems [11]. Here we report the first plasmonic polarizers in a cellulose-based LCs derived from natural materials and in a silica system that potentially confers greater device stability than its organic alternatives.

Scalability of nanofabrication is an important consideration for massive industrial production of solar energy photo-conversion and optical devices. Gold nanoparticles can be produced through numerous scalable syntheses [12] and are currently widely commercially available. Cellulose, the most abundant biopolymer on earth, is naturally found in wood and cotton as a blend of amorphous regions and rod-like crystallites with diameters of ~3–10 nm and lengths of 100–300 nm [13]. Facile sulfuric acid hydrolysis dissolves the amorphous regions leaving a charge stabilized colloidal suspension of rod-like crystallites that act as the building blocks of LC phases. Cellulose nanocrystals (CNCs) and mesoporous silica have been utilized as templates to assemble and synthesize nanoparticles [14–16], but the alignment of rod-like nanoparticles in these hosts has not been achieved. In this work, we first use these colloidal dispersions of CNCs to align co-dispersed GNRs of much shorter aspect ratio, with a longitudinal SPR peak within the visible optical spectrum, and then use the ensuing cellulose-gold colloidal nano-dispersion to obtain thin solid films with orientationally ordered organization of both cellulose and gold nano-colloids. Furthermore, we demonstrate that these structured films can be converted into mesoporous silica films decorated with aligned GNRs. Because of the combination of ordered plasmonic nanostructures, relatively large surface areas, and easy surface functionalization, the new composite mesoporous silica films may potentially offer many applications in optics, nanophotonics, biodetection, *etc*.

## 2. Results and Discussion

### 2.1. Cellulose Films with Aligned GNRs

The quality of alignment in films thicker than the size of nanorods is commonly characterized by the three-dimensional (3D) orientational scalar order parameter:

$$S=\frac{\langle 3\;{\text{cos}}^{2}\theta -1\rangle }{2}$$ (1)

where *θ* is the angle between the long axes of rods and the director **n** (describing local average orientation of these constituent rods). In our system, *S* is equal to the scalar order parameter corresponding to the longitudinal transition dipole moment. Therefore, the order parameter of GNRs can be deduced from the polarized optical transmission data by first determining the absorbance *A*^∥^ and *A*^⊥^ at the longitudinal SPR peak for polarizer orientations parallel and perpendicular to **n**, respectively and then using the expression

$$S=\frac{A^{∥}-A^{⊥}}{A^{∥}+2A^{⊥}}$$ (2)

**Figure 1.**
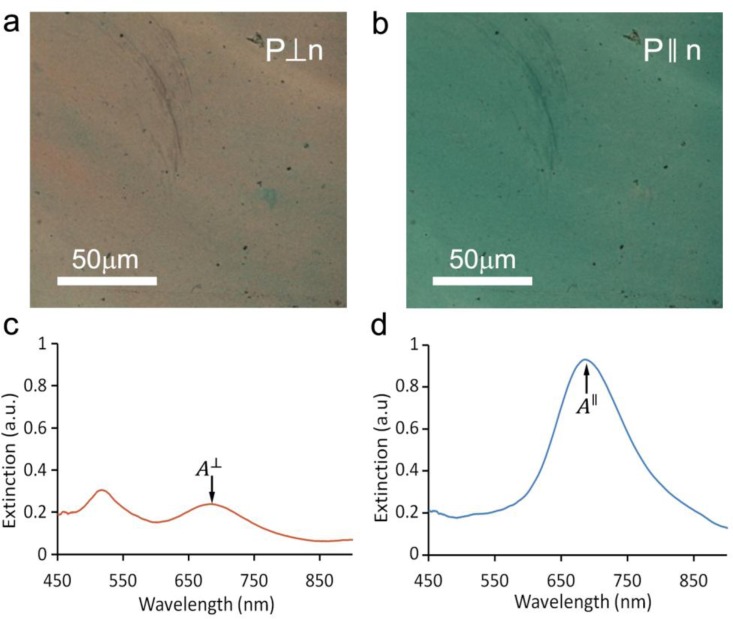
Cellulose films decorated with Gold nanorods (GNRs) produced by use of shear alignment of Cellulose nanocrystal (CNC)-GNR co-dispersions. (**a,b**) Bright field polarized microscopy images with analyzer aligned perpendicular (**a**) and parallel (**b**) to the director **n** show that nanorods are generally aligned along **n**, with absorption due to the transverse and longitudinal SPRs yielding pink and blue colors, respectively. (**c,d**) The extinction spectra of a thin dried film of CNCs and GNRs for linear polarizations perpendicular (**c**) and parallel (**d**) to **n**, confirming that the transverse and longitudinal SPRs with peaks at 525 nm and 680 nm, respectively, are significantly stronger in the perpendicular and parallel orientations, respectively.

We found that the simple by-hand shearing of CNC-GNR co-dispersions produced films of variable quality with some relatively large areas of uniform alignment (Figure 1a,b) with the GNRs having scalar order parameter *S* = 0.49, as determined from the optical extinction data (Figure 1c,d). This is comparable to the order parameter of ~0.35–0.65 for shear-oriented pure CNCs [17]. Previous studies, which optimized the mechanical shearing conditions, reported achieving films of highly aligned CNCs [18]. Therefore, one can expect that by controlling the shearing speed and concentration, it may be possible to reproducibly create even more highly aligned thin films with GNRs. If the shearing method is further optimized, it could potentially allow for large-scale production of uniform thin films composed of CNCs and GNRs on various thin substrates.

**Figure 2.**
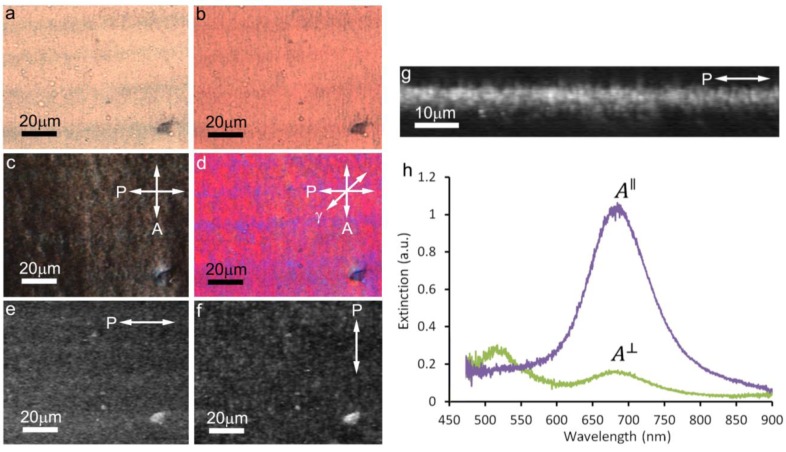
Aligned CNC films with aligned GNRs obtained by the gravity-assisted method. (**a,b**) Bright-field polarizing optical microscopy (POM) images with polarizations parallel (**a**), and perpendicular (**b**), to the director **n** show different colors associated with the two SPRs, respectively. POM images of a film between crossed polarizers (**c**) and with an inserted 530 nm retardation plate (**d**) with one polarizer parallel to **n**, allowing us to visualize imperfections in the alignment. (**e,f**) Two-photon luminescence (TPL) micrographs for polarization parallel (**e**) and perpendicular (**f**) to **n** demonstrate that GNRs are well dispersed within the film (no big aggregates observed within the uniform background due to the TPL signal from nanorods) align along **n**, with a stronger signal seen in (**e**). (**g**) The corresponding vertical cross-section for linear polarization of excitation light parallel to **n**. (**h**) Extinction spectra measured for polarization parallel and perpendicular to **n**.

However, we also find that the gravity-assisted alignment method could consistently produce higher quality aligned films without the need for any mechanization. This alignment is accomplished with a CNC-GNR liquid crystalline co-dispersion droplet stretched along the flow direction, becoming roughly semi-cylindrically-shaped with the contact lines parallel to the gravity-induced flow direction and the droplet’s thickness gradient perpendicular to it. Due to evaporation, the concentration of CNCs within a droplet is the highest along the edges, which causes a LC phase to first emerge at the boundary [19]. In the process of gravity-assisted alignment, there are flow forces, which tend to align **n** along the direction of flow [18], and LC elastic forces, which tend to align the liquid crystalline dispersion at the edges with **n** parallel to the contact line and orthogonal to the thickness gradient in the meniscus to avoid elastic-energy-costly director distortions [20]. Thus, in such an experimental deposition geometry, these two forces act in the same direction and augment each other in producing the desired unidirectional alignment of the LC. Consequently, the films tended to have the most uniform alignment parallel to both the contact line and the direction of flow, with the alignment quality being the best near the edges where both forces were present. Slow water evaporation retains this alignment relatively intact while producing orientationally-ordered solid films of CNCs. Bright field polarizing optical microscopy (POM) images shows that GNRs are generally aligned along **n** (Figure 2a,b). The dried films possess two well-defined extinction peaks, which were maximized when the incident light has linear polarization parallel or perpendicular to the uniformly aligned director **n**. The observed peaks in the obtained spectra correspond to longitudinal and transverse SPR effect, respectively, indicating a high degree of orientational ordering of plasmonic GNRs in the matrix of an aligned CNC-based thin film. Two-photon luminescence (TPL) images with the polarization parallel (Figure 2e) and perpendicular (Figure 2f) to **n** indicate that the GNRs are well aligned along the LC director of the CNC matrix. A cross sectional TPL image (Figure 2g) shows that this signal is strong over a vertical distance of ~2 μm, which indicates that the thickness of the film is on the order of 1 μm, since the vertical resolution is ~500 nm. The typical extinction spectra for a film with a 3D orientational order parameter of *S* = 0.67 are shown in Figure 2h, in which the longitudinal SPR wavelength (680 nm) is the same as that of GNRs in CNCs by shearing alignment. The scalar order parameter varied somewhat, depending on exact details of the thin film preparation, but was typically found to be as high as *S* = 0.67.

**Figure 3.**
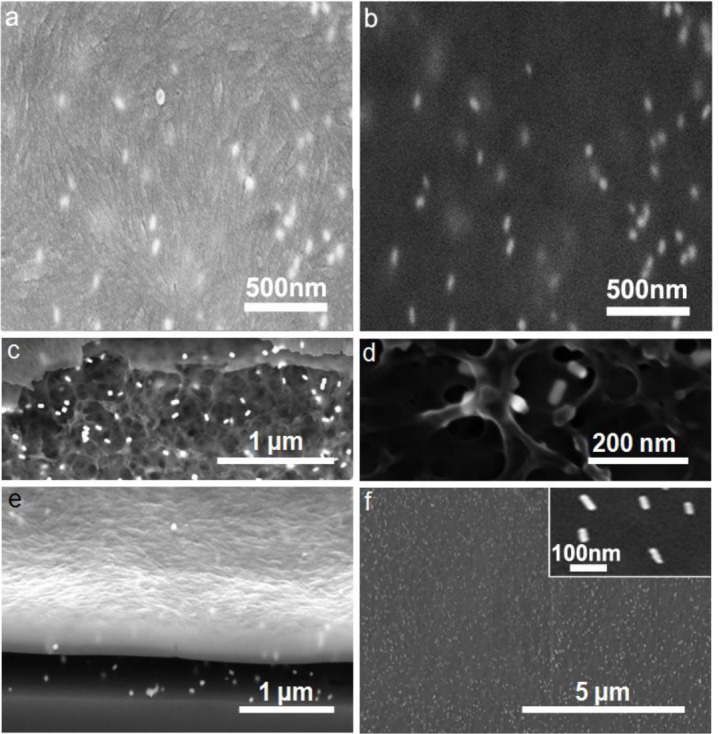
SEM imaging of thin films of CNCs with embedded GNRs (with a thin carbon coat to improve conductivity). (**a,b**) Co-located images obtained with (**a**) in-lens (IL), and (**b**) energy selective in-lens backscattered detector (ESB), detectors at 5 kV, allowing for simultaneous visualization of the unaligned CNC orientation field and the orientation of GNRs embedded in the film. (**c**) Everhart-Thornley (ET) imaging at low resolution and (**d**) IL imaging at high resolution reveal an interior network-like structure of the film at the site of a carbon film collapse induced by focused ion beam (FIB) in a nearby region. FIB allows for visualization of the cross-section of the film. ET imaging at 5 kV. (**e**) Tilt corrected for the 54° angle of the FIB milling, shows that the GNRs are distributed inside the bulk of the ~300 nm thick GNR film. The underlying glass substrate is visible in the bottom of the panel. (**f**) Low resolution ET imaging at 500 V demonstrates the long-range order of the film (no carbon layer is present). The inset shows the local orientation of GNRs.

To elucidate the nanoscale morphology of films, we used a combination of focused ion beam (FIB) milling and scanning electron microscopy (SEM) to probe the structural organization of CNCs and GNRs within the thin films. The images using the in-lens (IL) detector allow us to reveal the alignment of the CNCs (Figure 3a) while the energy selective in-lens backscattered detector (ESB) image emphasize the GNRs, which are aligned with the cellulose LC director field (Figure 3b). The interior structure of the film was revealed in an extended region where the conductive coating had collapsed due to nearby FIB milling. The interior structure of the CNC film can be understood as a nanoporous network-like arrangement with GNRs distributed throughout the bulk while exhibiting no positional but long-range nematic-like orientational ordering (Figure 3c,d). Furthermore, we used FIB to remove the top layer from a cross-sectional area to determine the thickness of the films, which was determined to be approximately 300–500 nm (Figure 3e). In the studied thin films, the unidirectional alignment could be seen extending over large distances over which the nanorods were uniformly dispersed (Figure 3f) and unidirectionally aligned (inset of Figure 3f).

### 2.2. Free-Standing Mesoporous Silica Films with Aligned GNRs

**Figure 4.**
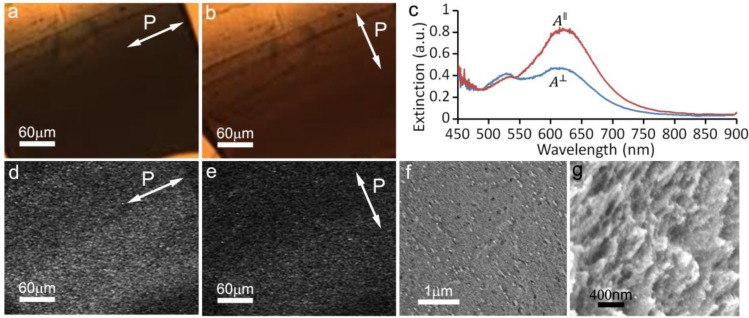
Mesoporous silica films containing aligned GNRs. (**a,b**) Bright field polarizing microscopy images with the polarization parallel (**a**) and perpendicular (**b**) to the director. (**c**) Absorption spectra of the film as measured with polarization parallel (red) and perpendicular (blue) to the director. (**d,e**) TPL images of GNRs taken with polarization parallel (**d**) and perpendicular (**e**) to the director, confirming alignment of the gold nanoparticles with their long axes on-average parallel to the director. The homogeneous TPL signal in the images confirms uniform distribution of GNRs. (**f,g**) SEM images of the mesoporous film showing (**f**) alignment of GNRs and (**g**) the porous morphology. To minimize charging, images (**f**) and (**g**) were acquired using ET at 1 kV and 2 kV primary beam acceleration voltage, respectively.

We also created thin free-standing films which consisted of only silica and GNRs by using a method previously reported in the literature [21], with certain modifications described below in the methods section. To obtain nematic-like ordering within the film we placed a drop of the solution on aluminum foil, where at the meniscus, the CNC LC experiences elastic forces which align it parallel to the receding contact line (in order to minimize elastic distortions and their free energy cost) [20]. Once the CNC-GNR films with nematic-like ordering were used as a replica for patterning nanoscale morphology of silica films, as described in the materials section, we found that the orientational ordering of GNRs persisted in the silica matrix upon removal of CNCs via the high-temperature treatment (Figure 4). Previous work [22] has shown that PVP capped GNRs are not stable at high temperatures and will transform into spheres in less than an hour at a temperature of 250 °C. However, we found that by using silica capped rods, the resultant films still possessed a well defined polarization-dependent SPR effect, indicating that silica capped GNRs maintained their rod-like structure. Using low-voltage SEM, we observed that the films had a mesoporous structure, which was consistent with what was previously observed for such films without GNRs [21]. GNRs tended to be evenly dispersed throughout the film, with a lower degree of alignment than in the cellulose films. The estimated scalar order parameter of GNRs that we could achieve by polarization-dependent extinction spectra in this case was *S* = 0.25. The reduced value of the orientational order parameter may be partially due to the high-temperature treatment known to affect gold nanoparticles [22]. The longitudinal plasmonic peak shifted from 680 nm (Figure 1d, 2h) to 630 nm (Figure 3c) due to a combination of two effects, the nanorods being deformed by heating and the silica having different dielectric properties than the cellulose. However, it is interesting that the alignment persisted upon removal of CNCs, providing a potentially useful approach for scalable fabrication of composite mesostructured films with orientationally ordered plasmonic nanoparticles in a silica matrix. These obtained hybrid thin films can be practically useful. The mesoporous silica surface, which encloses the GNRs, can be functionalized with various chemicals, and the unique absorption properties of the film could be used to study chemical reactions, or to create a catalyst for chemical reactions. The presence of orientationally-ordered plasmonic nanostructures in such films may provide the means of efficient use of light for controlling and guiding reactions and processes in various applications.

### 2.3. Discussion

Unlike their dichroic dye based counterparts, plasmonic polarizers allow for very precise control over the operational spectrum of a polarizer as well as the ability to polarize light of different wavelengths, including the ultraviolet and infrared parts of an optical spectrum [23]. The transverse plasmonic absorption is generally a function of the material used for the rod-like nanoparticles, with nickel having an absorption peak at 380 nm, silver absorbing at 420 nm, gold at 525 nm and copper at ~580 nm [23]. The longitudinal SPR is red shifted by an amount determined by the aspect ratio of the nanoparticle. Thus, a plasmonic polarizer potentially offers precise control of both the transverse and longitudinal device scale absorption across the electromagnetic spectrum, ranging from the ultraviolet through the visible and into the infrared [23]. Although plasmonic polarizers can be achieved by a number of different methods, such as stretching of polymer films with incorporated anisotropic plasmonic nanoparticles, our approach offers benefits of utilizing abundant cellulose-based nanomaterials and scalable self-assembly-based fabrication. The deposition of structured films from co-dispersions of anisotropic nanoparticles demonstrated with the example of metal nanorods and CNCs can be potentially extended to a variety of other technologically interesting nanoparticles, such as semiconductor-based quantum rods and discs as well as upconversion nanoparticles. Thus, a broad range of hybrid materials with different functionality can be potentially obtained using this approach.

Mesoporous silica films decorated with GNRs provide a more permanent structure that can potentially be incorporated into optical glasses. Furthermore, the mesoporous structure of these films allows for infiltration of solutions so that one can potentially use them in different forms of chemical and biological detection, exploring how exciting either the longitudinal or transverse plasmonic mode of a nanoparticle changes the catalytic activity. The pore size can be somewhat tunable by changing the source material of the cellulose, depending on the need of particular applications.

## 3. Experimental Section

### 3.1. Synthesis of Cellulose Nanocrystals

CNCs were synthesized using a known acid hydrolysis method [13,21]. Ten grams of bleached cotton was hydrolyzed in 150 mL of 65 wt.% sulfuric acid under continuous stirring in a 45 °C water bath. Every hour the mixture was sonicated for 5 min and a small amount of the solution was extracted and observed using POM to determine if the hydrolysis was complete. About 700 mL of deionized (DI) water was added to the completed reaction to quench the acid hydrolysis. Within a 24 hour period of time, the more concentrated dispersion of CNCs sedimented to a bottom layer, which constituted about 1/3 of the total volume, and the supernatant was removed. The cellulose was washed several times via redispersing in DI water, centrifugation, and removing the supernatant, which resulted in an increase of the pH to ~1. To further reduce the sulfuric acid content, the solution was placed inside of a dialysis tube (MWCO 12000–14000, Thermo Fisher Scientific Inc.) and dialyzed against DI water. The DI water was occasionally replaced, and the dialysis continued until the DI water maintained a constant pH, which normally took 2–3 days. After this, suspensions were filtered through Millipore filter with 3 μm holes. At this stage, the concentration of CNCs was normally 2–3 wt.% and was used in our experiments as described below.

### 3.2. Functionalization of GNRs

Cetyltrimethylammonium bromide capped GNRs with mean diameters and lengths of 20 nm and 50 nm respectively were synthesized in aqueous solution using a well-known method [24]. In order to protect against the acidic environment of the cellulose solution, the rods were recapped with methoxypolyethyleneglycol thiol (mPEG-SH, JemKem Technology) by a method described in [25]. Briefly, 300 μL of aqueous GNRs with optical density of ~100 and 300 μL of an aqueous 10 wt.% mPEG-SH solution were added to 10 mL of water. The solution was left at room temperature for 12 hours to ensure that the mPEG-SH had bonded to the surface of GNRs. The solution was centrifuged for 10 min at 10,000 rpm, and the supernatant was decanted. After this, the GNRs solution was re-dispersed using sonication. The capping procedure was repeated to ensure a complete coating of mPEG on the nanorods.

To incorporate the nanorods in mesoporous silica films using approaches that we will discuss below, the mPEG-capped GNRs were recapped with silica as described in [26]; 26 μL ammonium hydroxide (27% NH_3_ by weight) in 400 μL of H_2_O were mixed with 2 mL of ethanol. GNRs were then added until the solution had a slightly opaque blue color. After this, 0.24 μL of tetramethyl orthosilicate (TMOS) was added to the mixture. The mixture was then left overnight to react and, afterwards, the silica-capped GNRs were transferred into an aqueous solution by centrifugation and redispersion.

### 3.3. Shearing of Liquid Crystalline Co-Dispersions of CNCs and GNRs

The mPEG-capped GNRs dispersion was mixed with CNCs dispersed in water with a concentration ~3 wt.%, which was close to the critical concentration at which a chiral-nematic phase spontaneously forms (and was readily observed at drop edges and as water evaporated). To ensure that the nanorods were well dispersed, the sample was sonicated for at least 30 min. To shear the solution, a razor blade or pipet tip was held close a glass slide. A small amount of co-dispersed aqueous CNCs and GNRs was pipetted so that it formed a meniscus between the substrate and the glass slide. The razor blade or pipet tip was then moved back and forth at a slow speed. Each time it was moved back and forth, a thin coating of cellulose nanocrystals and gold nanorods was deposited. After water evaporation, the procedure was repeated again with another small amount of the solution. This was continued until approximately 50–150 microliters of solution had been deposited on a substrate with about one square centimeter area.

### 3.4. Gravity-Assisted Alignment of CNCs and GNRs

A glass substrate was placed at an oblique angle of 70–80° from the horizontal. A small drop of concentrated solution of mPEG capped GNRs and 3 wt.% CNCs co-dispersed in water was placed at the top of the slide and allowed to flow down under the action of gravity. After the solution reached the bottom, it was recovered with a pipet and again placed at the same location at the top of the slide. This process continued until the film reached a desired thickness, which was typically between 200 nm and 1 μm. The length of these aligned films is typically several inches long. We characterized the resultant film by use of a combination of focused ion milling and scanning electron microscopy techniques.

### 3.5. Free-Standing Mesoporous Films with Aligned GNRs

To create mesoporous silica films, we used a method developed by Shopowitz *et al*. [21], which we modified to achieve nematic-ordering by drying more quickly. 5 vol.% TMOS was added to an aqueous solution of 3 wt.% CNCs and dissolved by use of 30 min of sonication. Then an aqueous dispersion of silica capped GNRs was added slowly until the solution had a dark purple appearance. The solution was heated in an aluminum foil bowl on a hotplate with occasional agitation to prevent formation of a film of dried cellulose on top of the liquid. After the solution had dried, the ensuing dry films were peeled off the aluminum foil. To remove the cellulose from the films, the free-standing films were heated to 100 °C at a rate of 120 °C per hour and maintained at 100 °C for two hours. Then, the samples were baked at 540 °C for eight hours, and subsequently ambient-air-cooled down to the room temperature. This thermal treatment assured that all CNCs were eliminated from the mesoporous films obtained through the above-described preparation procedure. The size of these silica mesoporous films with aligned GNRs is on the scale of millimeters.

### 3.6. Nonlinear Optical Luminescence Imaging

TPL imaging was performed on an inverted IX-81 Olympus microscope using 850 nm excitation light from a tunable Ti:Sapphire oscillator (Chameleon Ultra II, Coherent) with 140 fs pulse width and 80 MHz repetition rate at an average power < 1 mW in the sample plane. A 60× oil objective with NA = 1.42 was used for epi-detection of TPL within a 400–700 nm range (selected by an appropriate interference filter) by a photomultiplier tube (H5784-20, Hamamatsu). To probe spatial patterns of orientation, the linear polarization direction of the excitation light was controlled using a half-wave retardation plate placed immediately before the objective. The experimental setup and technique are described in details elsewhere [2].

### 3.7. Focused Ion Beam Milling and Scanning Electron Microscopy

The nano-scale structure and morphology of the resultant films of CNCs and silica with GNRs were characterized using a combination of FIB milling and SEM by means of a FIB-SEM (Auriga, Carl Zeiss). The studied films were poor electrical conductors and, therefore, a variety of charge reduction techniques were employed to create images of sufficient resolution without distortion and artifacts. In the case of mesoporous silica, the imaging was performed for free-standing films, and low resolution imaging of long range order in CNC films was obtained as deposited on glass slides. To eliminate the effects of charge accumulation for these samples, an acceleration voltage close to the secondary emission crossover point was chosen [27]. However in the case of high resolution and bulk characterization of CNCs with GNRs, a charge dissipation layer of carbon (~50 nm in thickness) was sputtered over the film and glass coverslip. To clearly identify the GNRs, CNCs or mesoporous silica, the energy of secondary electrons collected was controlled by choice of detector. Three detectors were used: an IL, a standard Everhart-Thornley (ET), and ESB to choose from low energy-high angle, mixed energy-angle insensitive, and high energy-high angle secondary electrons, respectively. Low energy-high angle secondary electrons reveal the surface structure of films, whereas higher energy secondary electrons provide material contrast emphasizing GNRs. The interior structure of the film was revealed by milling the sample with the FIB, and imaged either at the site of the milling or at a location where the sputtered carbon film had collapsed.

## 4. Conclusions

We have demonstrated that rod-like cellulose nanocrystals forming liquid crystalline phases can confer alignment to gold nanorods within thin solid films prepared by several different deposition methods. We have also demonstrated scalable fabrication of thin mesoporous films of silica decorated by orientationally ordered anisotropic gold nanoparticles. These simple, reproducible methods for fabrication of plasmonic nanostructured thin films with orientational ordering and polarization sensitivity of optical properties demonstrate the power of self-assembly to create unique optical characteristics in thin composite films. The achieved combination of ordered nanoscale morphology in both silica and organic cellulose-based films, along with the long-range oriented metal nanostructures, are of great interest for renewable energy, optical, catalytic and anti-counterfeiting technologies.

## Disclaimer

Contribution of the U.S. Department of Commerce is not subject to copyright in the U.S; licensee MDPI, Basel, Switzerland. Certain commercial equipment, instruments or materials are identified in this document. Such identification does not imply recommendation or endorsement by the National Institute of Standards and Technology.
